# Quality of life and disease activity assessment in patients with chronic urticaria

**DOI:** 10.1186/1939-4551-8-S1-A91

**Published:** 2015-04-08

**Authors:** Gabriela Andrade C Dias, Fernanda Cunha Coelho, Carolina Pennaforte, Fabiana Caputo, Anna Carolina Nogueira Arraes, Denise Lacerda Pedrazzi, Maria Ines Perelló, Eduardo Costafsilva, Priscilla Filippo Alvim De Minas Santos

**Affiliations:** 1 State University of Rio De Janeiro, Brazil; 2 Brazillian Association of Allergy and Immunology, Brazil

## Background

Chronic urticaria (CU) is a debilitating disease that affects patients’ quality of life (QoL) and the only questionnaire developed specifically to CU is the *Chronic Urticaria Quality of Life Questionnaire* (CU-Q_2_oL). The aim of this study was to evaluate the QoL of patients with CU and to correlate it with disease activity.

## Methods

The Brazilian Portuguese version of the CU-Q_2_oL was self-administered in 96 adults with CU treated at the outpatient clinic of a university hospital. Disease activity was assessed using the Urticaria Activity Score (UAS). The following characteristics were also studied: age, gender, type of urticarial and duration of disease. ANOVA was used to compare the results of CU-Q_2_oL between the different groups. The relationship between the CU-Q_2_oL total score and dimensions and the UAS score was assessed by Pearson correlation coefficient.

## Results

The study population comprised 85% women, with a median age of 46.5 years (IQR: 31-58.5) and median disease duration period was 19 months (IQR: 9.25-60). The main diagnosis was chronic spontaneous urticaria (CSU) (55.2%), with 21.9% associated with physical urticaria (PU); 18.8% had chronic autoimmune urticaria (CAIU), with 11.4% associated with PU, 27.1% had PU alone and 53.1% presented dermographic urticaria. Mean UAS score was 1.52±1.73 (0-6). The total CU-Q_2_oL mean score was 33.39±21 (0-100) and dimension I (sleep/mental state/feeding) had the greatest impact on QoL. The items with the highest mean score were about itching (59) and nervousness (58) and the lowest were about eye edema (34) and sports activities’ limitations (35). The analysis of variance showed that women had greater impairment of quality of life in the dimensions I and III (edema/limitations/appearance) (*p*=0.02; *p*=0.01). Patients with CAIU and PU are more affected in total score and in all dimensions (*p*=0.005; *p*=0.008; *p*=0.04; *p*=0.008). The CU-Q_2_oL was moderate correlated with UAS (r=0.45) (*p*<0.000) and was able to discriminate between patients with different degrees of disease severity.

## Conclusions

CU patients’ have severely impaired QoL and a significant emotional burden. This study showed that women and patients with CAIU associated with PU had the greatest QoL impact. CU-Q_2_oL and UAS are important instruments not only in research, but also to evaluate treatment outcomes and must be used in clinical practice.

